# Influence of valve size on the hemodynamic performance of a tissue-engineered valved conduit in pulmonary position 

**DOI:** 10.3389/fbioe.2025.1629362

**Published:** 2025-08-26

**Authors:** Lorenzo Ferrari, Martijn Cox, Dominik Obrist

**Affiliations:** ^1^ ARTORG Center for Biomedical Engineering Research, University of Bern, Bern, Switzerland; ^2^ Xeltis BV, Eindhoven, Netherlands

**Keywords:** heart valve, tomo-PIV, in-vitro, shear stresses, shake-the-box

## Abstract

**Introduction:**

Tissue Engineering (TE) uses resorbable polymers to promote *in-situ* cellular growth, transforming the implant into a living valve. This study characterizes the three-dimensional flow field around TE valved conduits of varying sizes using a pulse duplicator with tomo-PIV imaging.

**Methods:**

Three Xeltis Pulmonary Valve (XPV) conduits (16, 18, and 20 mm) were tested under pulmonary conditions at a cardiac output of 5 L/min. Flow velocities, trans-valvular pressure gradients (*TVPGs*), effective orifice areas *EOAs*, mean and turbulent kinetic energies (*mke* and *tke*), and viscous shear stresses were measured proximal and distal to the valves.

**Results:**

Peak bulk velocity was 0.5, 0.4, and 0.3 m/s, with local peak velocities reaching 2.3, 1.9, and 1.4 m/s upstream and 3.6, 3.1, and 2.5 m/s in the jet downstream of XPV16, XPV18, and XPV20, respectively. Respective *EOAs* were 1.02, 1.25, and 1.57 cm^2^. The flow field proximal to the valve conduits did not show any significant perturbations and *tke* was one order of magnitude lower than *mke*. As the flow passed the valve, *mke* increased by 152%, 175%, and 218% for XPV16, XPV18, and XPV20, respectively, while *tke* increased by 62%, 138%, and 161%. The respective probability of encountering elevated shear stresses (>10Pa) was 6%, 2%, and less than 1%.

**Discussion:**

This work provides the first *in-vitro* experimental assessment of the XPV valve, along with an exploration of how valve size affects its hemodynamic performance. Results confirm that for a given hemodynamic condition, larger valves exhibit better performance showing lower flow velocities, *TVPGs*, kinetic energies, and stresses, along with higher *EOAs*.

## 1 Introduction

Anomalies of the right ventricular outflow tract (RVOT) cover a wide range of structural cardiac malformations. Accounting for approximately 20% of all congenital heart diseases (CHDs), RVOT reconstruction is the most common procedure in congenital cardiac surgery (Allen, 2008; Costello et al., 2014; Walters et al., 2000). The reconstruction can be performed with valved grafts such as homografts and xenografts. However, these implants commonly result in mid-to long-term complications including stenosis induced by increased narrowing and degradation with or without calcification (Bonetti et al., 2019; Grubitzsch et al., 2013; Lis et al., 2003). Moreover, current solutions are unable to accompany the physiological development of the younger patients, necessitating repeated surgery (DiBardino and Jacobs, 2014). Tissue engineering (TE) is a promising emerging technique to promote endogenous tissue restoration (ETR) (Bouten et al., 2018; Wissing et al., 2017; Fioretta et al., 2021). The idea is to use resorbable polymeric constructs to trigger cellular infiltration, turning the implant into a living functional valve (Smits and Bouten, 2018). An emerging TE valve is the Xeltis Pulmonary Valved Conduit (XPV, Xeltis, Eindhoven, Netherlands), designed to promote ETR. The XPV is produced through electrospinning and consists of two distinct supramolecular polymers: a polycaprolactone-based UPy polymer forming the conduit, and a polycarbonate-based UPy polymer used for fabricating the leaflets. (Mes et al., 2022). The conduit is designed to be reabsorbed *in vivo* by oxidative oxygen species and enzymatic degradation (Bennink et al., 2018). To assess the feasibility and optimize its design for human application, several studies have been carried out. The valve was surgically implanted for short term evaluations in sheep models showing safety and functionality (Bennink et al., 2018). The information collected from this study was utilized to improve the graft design and the two final valve designs were recently implanted for clinical trials. The first report on early outcomes shows positive results on right ventricular outflow tract reconstruction in pediatric patients (Morales et al., 2021). Despite these promising results, the inflammatory and regenerative mechanism following *in vivo* remains unclear (Duijvelshoff et al., 2021). To better understand this phenomenon, De Kort et al. (2021) evaluated these phenomena in a retrospective study on the explanted material from the previously reported clinical study on sheep. The study demonstrated significant spatial heterogeneity in cellular distribution across the valve leaflet, suggesting that local hemodynamic loads play a crucial role in influencing cellular repopulation and tissue regeneration. Furthermore, effects of turbulent flow have been associated to endothelial cell turnover (Davies et al., 1986), blood trauma (Holmes and Mack, 2017), prosthetic valve calcifications (Tsolaki et al., 2023) and up to an 20%–30% increase in the hydraulic resistance (Becsek et al., 2020). This highlights the importance of understanding the local hemodynamics to guide improvements in graft design and functionality, ultimately enhancing their regenerative potential and clinical performance.

The aim of this study is to quantitatively assess how vlve size affects the flow field around heart valve replacements by analyzing the flow field in different sizes of XPVs. Within this study, we characterize the *in-vitro* flow field by means of tomographic particle imaging velocimetry (tomo-PIV) to understand the effect of hemodynamics on valve performance, which contributes to valve design optimization (Scarano, 2012). Furthermore, we can relate hemodynamics with thrombotic mechanics as well as leaflet deterioration and calcification of the biological leaflet tissue (Tsolaki et al., 2023; Pietrasanta et al., 2022; Hasler and Obrist, 2018; Hasler et al., 2016; Ferrari and Obrist, 2024; Chen and Dasi, 2023). To the authors’ best knowledge, this is the first time a detailed experimental flow field measurement has been done for a TE valve in pulmonary position. This work provides high-resolution insights into flow characteristics around XPV valves, exploring the effects of different valve sizes.

## 2 Materials and methods

A multi-view imaging system for tomo-PIV was employed to perform *in-vitro* measurements of the three-dimensional flow field in the aortic root. The same configuration was previously used to study surgical and transcatheter heart valves (Pietrasanta et al., 2022; Hasler and Obrist, 2018; Hasler et al., 2016; Ferrari and Obrist, 2024). For the present study, the setup was adapted for TE valved conduits and to mimic the hemodynamic conditions in the right side of the heart.

### 2.1 Sample preparation

**FIGURE 1:**
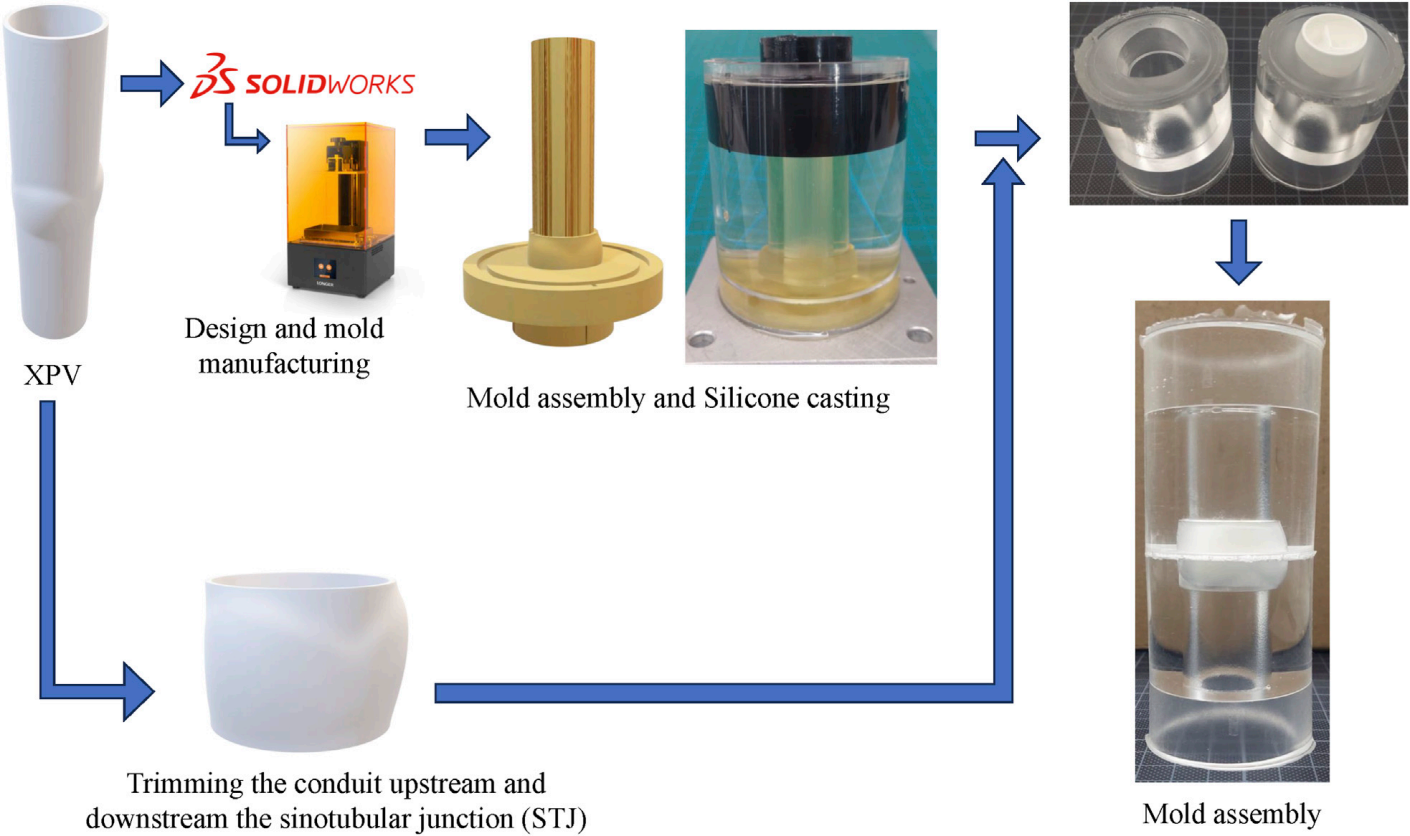
Schematic of the sample preparation for the XPVs conduit. The technical drawings of each model of XPV are used to 3D-print the negative of the upper and lower half of each conduit. While the sinus portion accounts for the wall thickness, the straight segments are representative of the inner lumen. The XPV valve is then trimmed off the extremities and glued between the two-halves of the phantom using additional silicone.

A total of three XPV conduits with nominal diameters of 16, 18, and 20 mm were tested in pulmonary position. The XPV conduits consisted only of the polymeric scaffold, which were tested without populating the matrix with any cellular component. A single specimen was tested per valve dimension. The steps required to prepare a test sample for tomo-PIV measurements are illustrated in Figure 1. The XPV conduits were adjusted to provide optical access to the flow field upstream (proximal) and downstream (distal) of the valve. To this end, the conduits were cut at the levels of the annulus and of the sinotubular junction (STJ) and glued between two-halves of a silicon phantom of the aortic root. The phantom accurately represented the inner lumen of the removed conduit portions. This configuration allowed the measurement of the flow fields 3 cm upstream of the valve annulus (inflow) and downstream of the STJ.

The phantoms for the 16, 18, and 20 mm conduits were fabricated by mold casting, curing silicone (ELASTOSIL 601, Wacker Chemie AG, Germany) for 24 h at room temperature. This silicone matched the refractive index of the blood-mimicking testing fluid (Geoghegan et al., 2012). Additional silicone was prepared as glue to connect the components of the samples.

### 2.2 Test system and hemodynamic configuration

**FIGURE 2:**
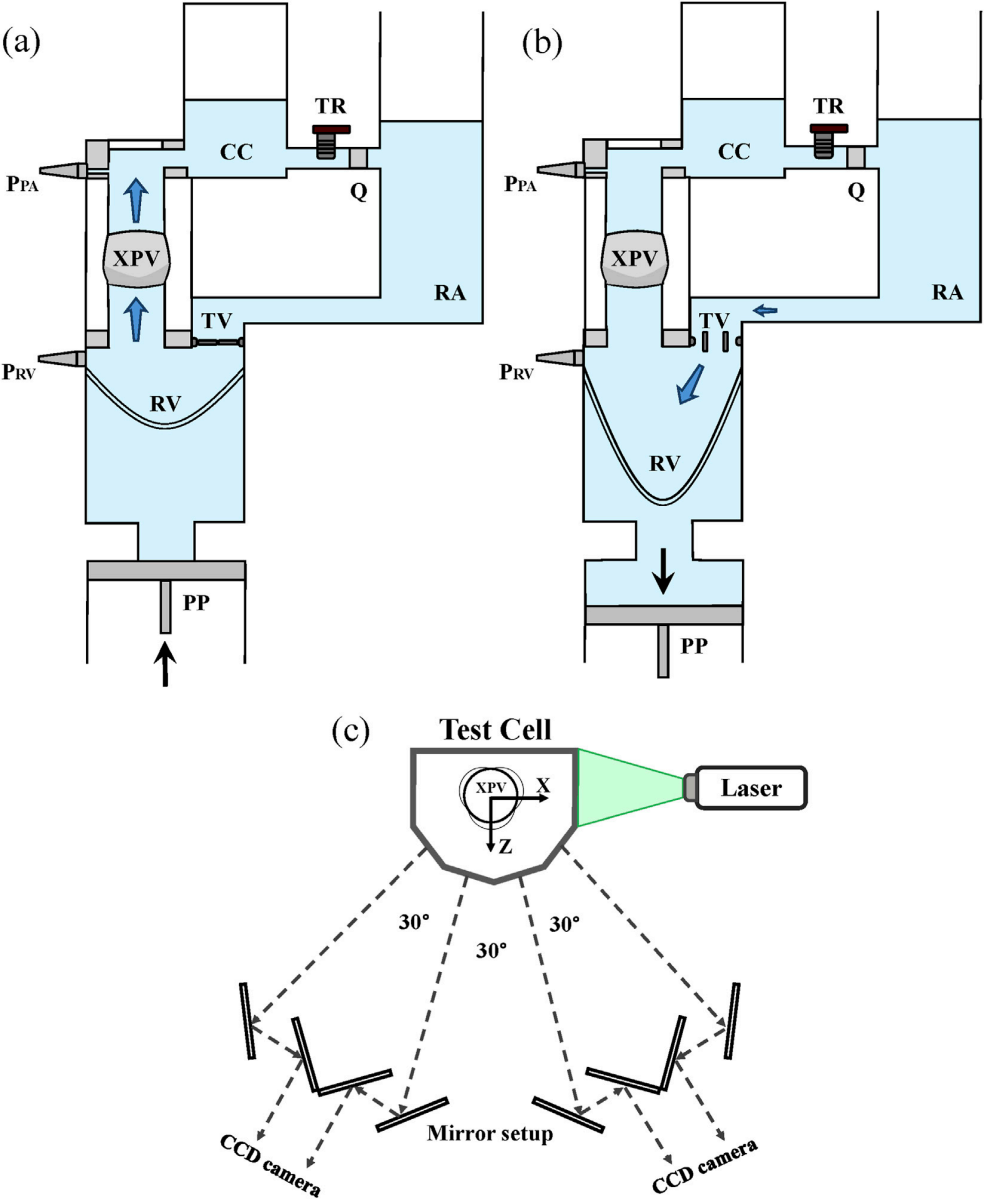
The pulse duplicator and its components during the systolic phase (XPV open - **(a)**) and the diastolic phase (XPV closed - **(b)**). Flow sensor (Q) and ventricular and pulmonary pressure sensors (PRV and PPA) are shown in the figure. Scheme of the optical system for tomo-PIV measurements **(c)**.

Details of the testing system can be found in previous work by the authors (Ferrari and Obrist, 2024). In brief, the pulse duplicator consists of a system of rigid chambers with tunable compliance (CC) and resistance (TR) used to mimic physiological conditions upstream and downstream the valve. A computer-controlled piston pump (PP) connected to the system allows to adjust pulsatile conditions and flow rate in the system. When the PP moves forward, the membrane in the right ventricle (RV) is compressed. The forward motion of the piston is representative of the systolic phase of the cardiac cycle. At this point, the ventricular pressure increases, overcoming the pressure in the pulmonary artery (PA) and in the right atrium (RA) such that the tested XPV opens while the tricuspid valve (TV) closes. The refractive index matched (RIM) testing fluid, is pushed from the ventricle through the XPV, the CC, and the TR into the atrium. During diastole, the piston retracts, inducing the closure of the XPV and the opening of the TV. The flow motion, induced by the periodical movement of the piston, allows for a dynamic opening and closing of the tested valve under physiological pressure conditions. The RIM fluid is then filling the ventricle, concluding the cardiac cycle. A schematic of the test system with its components is shown in Figure 2.

The RIM fluid consists of a solution of water/glycerol/NaCl (Sigma Aldrich Corporation, St. Louis, MO, United States) with a weight ratio of 0.494:0.340:0.166, resulting in a density of $\rho_{f}$ = 1,200 kg/m^3^ and a kinematic viscosity ν = 4.7 mm^2^/s. The RIM fluid was designed to match the refractive index of the silicone used during sample preparation to minimize optical distortion. Cardiac output was measured with an ultrasonic flow probe (Transonic Systems Inc., Ithaca, NY, United States) while Baumer-PBMN pressure sensors were located upstream and downstream of the straight segment of the tested XPV. Signals were sampled at 1 kHz.

**FIGURE 3:**
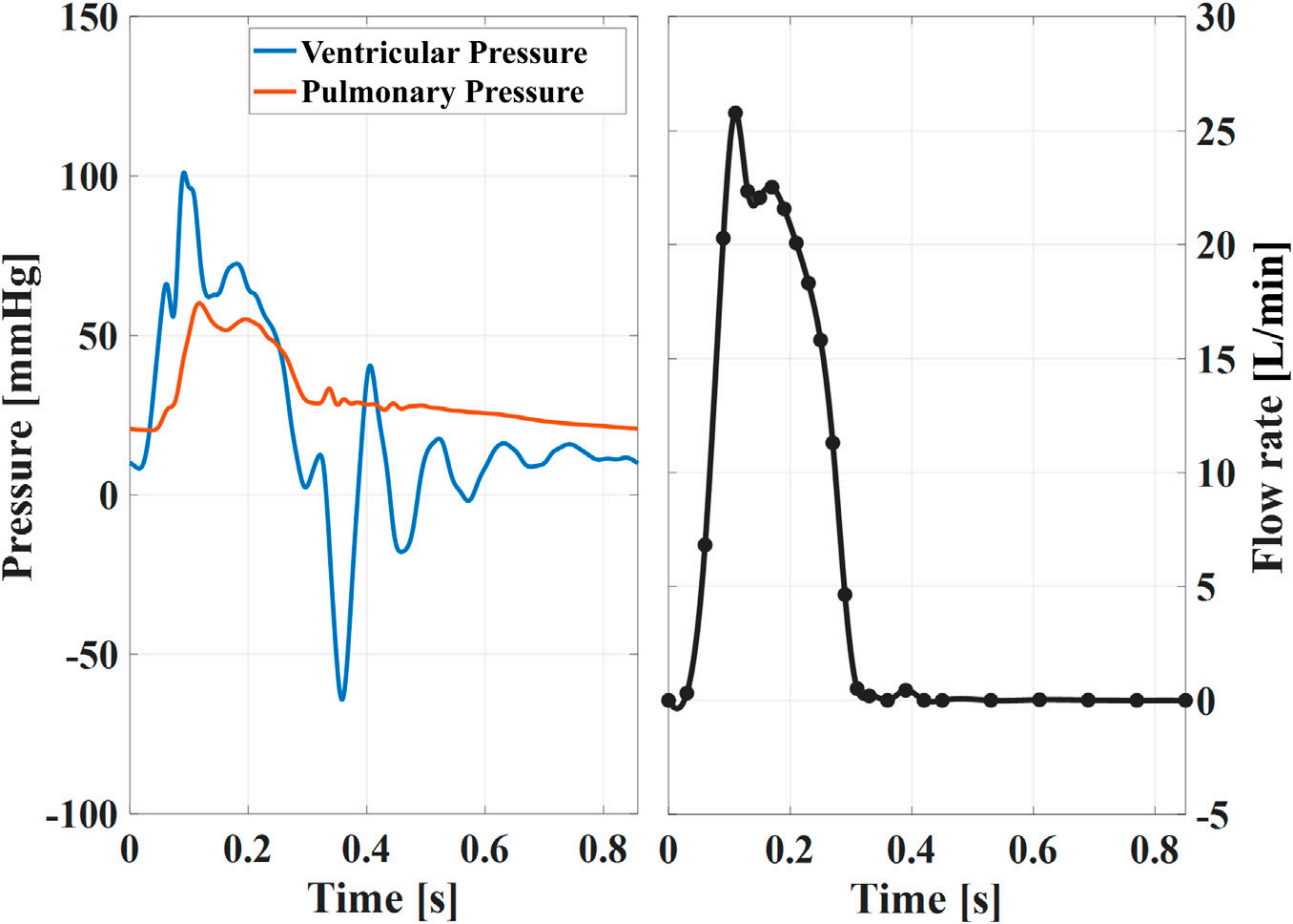
Example of instantaneous pressures (left) and ensemble averaged flow rate (right) for the XPV18 valve. These curves are almost identical among different sizes, thus representative of the waveforms XPV16 and XPV20. The black dots indicate the time points tφ when tomo-PIV data was acquired.

Each valve was tested for a cardiac output (*CO*) of 5 L/min. The heart rate was set to 70bpm and the systolic duration to 35% of the cardiac period. Representative pressure and flow conditions are shown in Figure 3. For each tested valve, the flow rate was prescribed while the ventricular and pulmonary pressures were used as checkpoints for physiological hypertensive conditions (Figure 3). This configuration was chosen to test the valve in the upper range of the corresponding pediatric category based on the valve size, which corresponds to children in an age spanning between 5 and 13 years old (Yoganathan et al., 2013).

### 2.3 Tomo-PIV reconstruction

Details of the multi-view imaging hardware can be found in Ferrari and Obrist (2024). Furthermore, a Two-Pulse 3D particle tracking with Shake-The-Box (STB) was used to analyze the image data (Novara et al., 2023). This algorithm is based on an iterative particle reconstruction algorithm and it is used for Lagrangian particle tracking in high-density seeded flow (Schröder and Schanz, 2023). The Eulerian velocity field is then reconstructed as the weighted average of the trajectories contained in the voxel over *N* consecutive beats. The STB algorithm yields a lower number of undetected particles together with a lower number of wrongly detected particles (ghost particles) and lower average error in the particle detection compared to a classical SMART algorithm (Schanz et al., 2016). Camera and volume calibration, image preprocessing, volume reconstruction, and the computation of the 3D instantaneous velocity vector fields with STB were performed in DaVis 10 (LaVision GmbH, Göttingen, Germany). Pulse delay was set to 800 *µ*s during diastole and 400 *µ*s during acceleration and deceleration phases for both conditions, while it was reduced to 200 *µ*s for peak systole, respectively. With an interrogation volume of 36 voxels (1.28 mm) and 75% correlation overlap, the final spatial resolution of the velocity field is given in Equation 1:

$$\delta =36\;\cdot \;\left(1-0.75\right)\;\cdot \;1\;vx\approx 0.32\;mm$$ (1)

### 2.4 Experimental protocol

A series of *N* = 200 flow field measurements were performed at different phases (*φ*) of the cardiac cycle, with images obtained at time *t* = *t*_*φ*_ + *nT*, where *n* = 1, 2, . . ., *N* and *T* represents the period of the heart cycle. The number of repetitions were chosen based on previous calibration of the system to ensure ±2% convergence of the measurement in all domain (Ferrari and Obrist, 2024). Prior to data acquisition, each XPV was exposed to ≈30 min operating cycles to ensure steady-state conditions in the viscoelastic behavior of the leaflets. After removing the static background from each image, a mask was applied to remove any intensity outside of the region of interest (ROI). Finally, the particle intensity was normalized over all frames and across the whole field of view. For each time instance, a subset of 10 images was used for particle tracking without predictor, with a wide search radius. The histogram of each velocity component was plotted to assess the main flow direction. This information was used as a predictor for a second tracking, increasing the number of images to 50. At this point, the ROI was divided into sub-volumes of 40 voxels, and the binned velocity field was computed in each voxel as ensemble average with a 75% correlation overlap. The ensemble-averaged velocity, together with a smaller search radius, was finally used as a predictor to reconstruct the trajectories over the whole *N* = 200 recordings. This process was repeated for each time instance. The final mean Eulerian velocities $\bar{v}\left(x\right)$ were computed in each voxel as a Gaussian weighted average using the particle tracks of all *N* images per phase. A total of 25 phases were chosen with increasing density in proximity of the systolic peak, such as:

$$t_{\varphi }=\left[0.00,0.03,0.06,0.09,0.11,0.13,0.15,0.17,0.19,0.21,0.23,0.25,0.27,0.29,0.31,0.33,0.36,0.39,0.42,0.45,0.53,0.61,0.69,0.77,0.85\right]s$$

While the methodology proposed by Hasler et al. was implemented to reduce systematic uncertainties (Hasler and Obrist, 2018; Hasler et al., 2016), previous work of the authors quantified the normalized uncertainties of the setup below 1% in all domain when operating the system at 5 L/min (Ferrari and Obrist, 2024).

The Reynolds and Womersley numbers to characterize the flow field were defined as shown in Equations 2 and 3:

$$Re=\frac{{4\;Q}_{\max}}{D_{a}\pi \nu }$$ (2)

$$Wo=\frac{D_{a}}{2}\sqrt{\frac{2\pi }{T\nu }}$$ (3)

where *Q*_max_ was the peak systolic mean flow rate, and *D*_*a*_ = 16, 18, or 20 mm was the respective diameter of the tested XPV.

### 2.5 Data analysis

The resulting velocity fields $v=\left[v_{x},v_{y},v_{z}\right]$ were separated according to the Reynolds decomposition into fluctuating ( $v^{\prime }$ ) and mean ( v ) components (Equation 4) (Pope, 2000):

$$v=\bar{v}+v^{\prime }$$ (4)

The mean velocities $\bar{v}\left(x\right)$ were computed according to Equation 5 by phase-averaging the instantaneous velocity field:

$$\bar{v}\left(x,t_{\varphi }\right)=\frac{1}{N}\;\sum_{n=1}^{N}v\left(x,t_{\varphi }+\left(n-1\right)T\right)$$ (5)

The mean flow rate was extracted from the reconstructed velocity profile according to Equation 6:

$$\bar{Q}={\bar{v}}_{y}\cdot A$$ (6)

where *A* is the cross-sectional area of the aorta and $\bar{v}\left(y\right)$ is the mean streamwise velocity. The bulk velocity is then defined as $V_{y}=\bar{Q}/A$ .

Flow rates were computed at multiple streamwise locations (with a spacing of 0.3 mm, matching the voxel resolution) between the STJ and the distal end of the ascending aorta.

The effective orifice area (*EOA*) was calculated from flow and pressure in Equation 7 according to standard (ISO 5840-1) as:

$$EOA=\frac{Q_{RMS}}{51.6\sqrt{\frac{{∆P}_{mean}}{\rho }}}$$ (7)

where *Q*_*RMS*_ (in mL/s) is the root-mean-square of the flow computed during the positive period of pressure difference and *∆P*_*mean*_ (in mmHg) is the mean systolic transvalvular pressure gradient (*TVPG*).

The mean kinetic energy (*mke*) [J/kg] and the turbulent kinetic energy (*tke*) [J/kg] were computed from the three components of the mean velocity $\left[v_{x},v_{y},v_{z}\right]$ and the velocity fluctuations $\left.{\left[v\right.}_{x}^{\prime },v_{y}^{\prime },v_{z}^{\prime }\right]$ as shown in Equations 8 and 9:

$$tke=\frac{1}{2}\left(\bar{v_{x}^{\prime 2}}+\bar{v_{y}^{\prime 2}}+\bar{v_{z}^{\prime 2}}\right)=\frac{1}{2}\;v_{rms}^{\prime 2}$$ (8)

$$mke=\frac{1}{2}\left.\left({\bar{v}}_{x}^{2}+{\bar{v}}_{y}^{2}+{\bar{v}}_{z}^{2}\;\right)\right.=\frac{1}{2}\;{\left|\bar{v}\right|}^{2}.$$ (9)

Finally, the maximum viscous stress τ (Pa) (Equation 10) was estimated from the minimum and maximum eigenvalues, $\lambda_{\min,\max}$ , of the strain-rate tensor ( $S_{ij}$ ) (Equation 11):

$$\left.\tau =µ\;\right|\lambda_{\max}\left(S\;\right)-\lambda_{\max}\left(S\;\right)|,$$ (10)

With

$$S_{ij}=\frac{1}{2}\;\left(\frac{{\partial v}_{i}}{{\partial x}_{j}}+\frac{{\partial v}_{j}}{{\partial x}_{i}}\right)$$ (11)

## 3 Results

### 3.1 Flow characteristics

**FIGURE 4:**
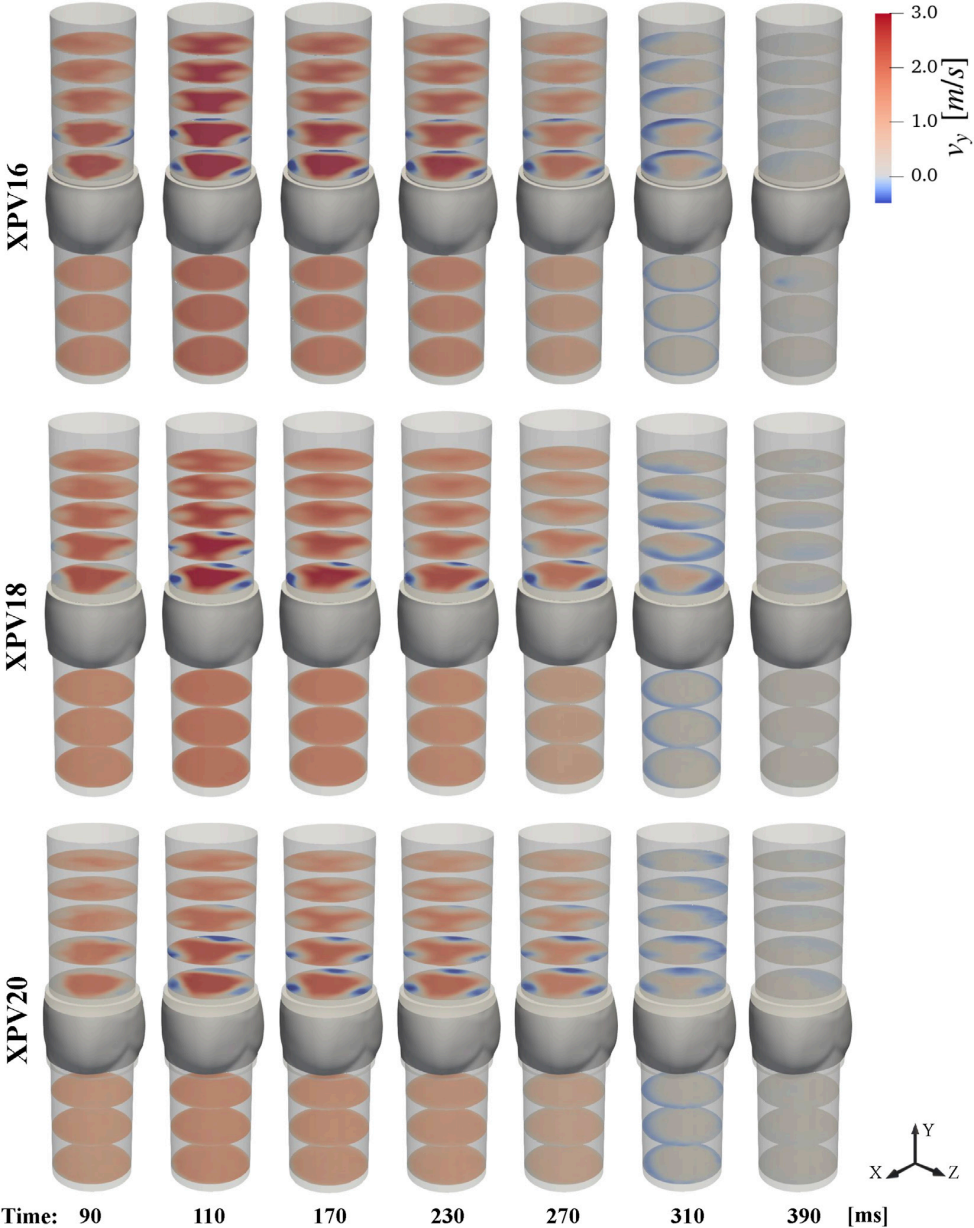
Temporal evolution of the mean streamwise velocity ${\bar{v}}_{y}$ .

Upstream of the XPV valves, we observed fully developed axial flow without any large coherent structures disturbing the flow. The velocity profiles did not change noticeably in streamwise direction. The flow rates computed from reconstructed velocities matched the prescribed values and varied only little between different experiments. As expected, the bulk velocities decreased with increasing valve size. Accordingly, the Reynolds numbers at peak flow were smaller for the larger valves. In contrast, the Womersley numbers were slightly higher for the larger valves. During diastole, all three valves were tightly sealed as indicated by the absence of negative streamwise velocities upstream of the leaflets, as it can be appreciated in Figure 4 for $t_{\emptyset }$ = 310 and 390 ms.

The pressure drops across the valves (transvalvular pressure gradients, *TVPGs*) decreased significantly with valve size, while *EOA* increased simultaneously. Considering the peak velocities, the *TVPGs* were below relevant values requiring interventions and aligned with previously reported clinical data where the XPV size 16 and 18 were implanted in children (Morales et al., 2021; Heaton et al., 2025). Hemodynamic parameters are summarized in Table 1.

**TABLE 1:** Hemodynamic parameters for XPV16, XPV18, and XPV20. The mean standard deviation of TVPG over the 200 consecutive beats collected at each phase of the cardiac cycle, for a total of 5,000 beats, is given in parentheses.

Parameter	XPV16	XPV18	XPV20
Peak bulk velocity (m/s)	0.50	0.42	0.32
95th percentile of velocity upstream (m/s)	2.3	1.9	1.4
95th percentile of velocity downstream (m/s)	3.6	3.1	2.5
*TVPG* (mmHg)	25.5 (0.6)	19.7 (0.5)	12.1 (1.0)
*EOA* (cm^2^)	1.02	1.25	1.57
*Re*	≈3, 500	≈3, 000	≈2, 500
*Wo*	10.0	11.3	12.5

Downstream of the valves, the flow profile was characterized by a central triangular jet confined by three regions of slow retrograde flow along the wall above each leaflet. The evolution of the streamwise velocity throughout the pulse cycle is shown in Figure 4. Considering the 95th percentile peak value at peak systole, the velocities at the valve orifice decreased with valve size. The velocities in the central jet increased with respect to the inflow by 58.8%, 65.9%, and 78.4% for XPV16, XPV18, and XPV20, respectively, indicating that the flow in the larger valves experienced higher acceleration. Finally, the triangular footprint of the valve persisted farther downstream for the smaller XPV, whereas the triangular structure dissipated more rapidly for XPV20 (see, e.g., Figure 4 at 110 ms).

### 3.2 Mean and turbulent kinetic energy

**FIGURE 5:**
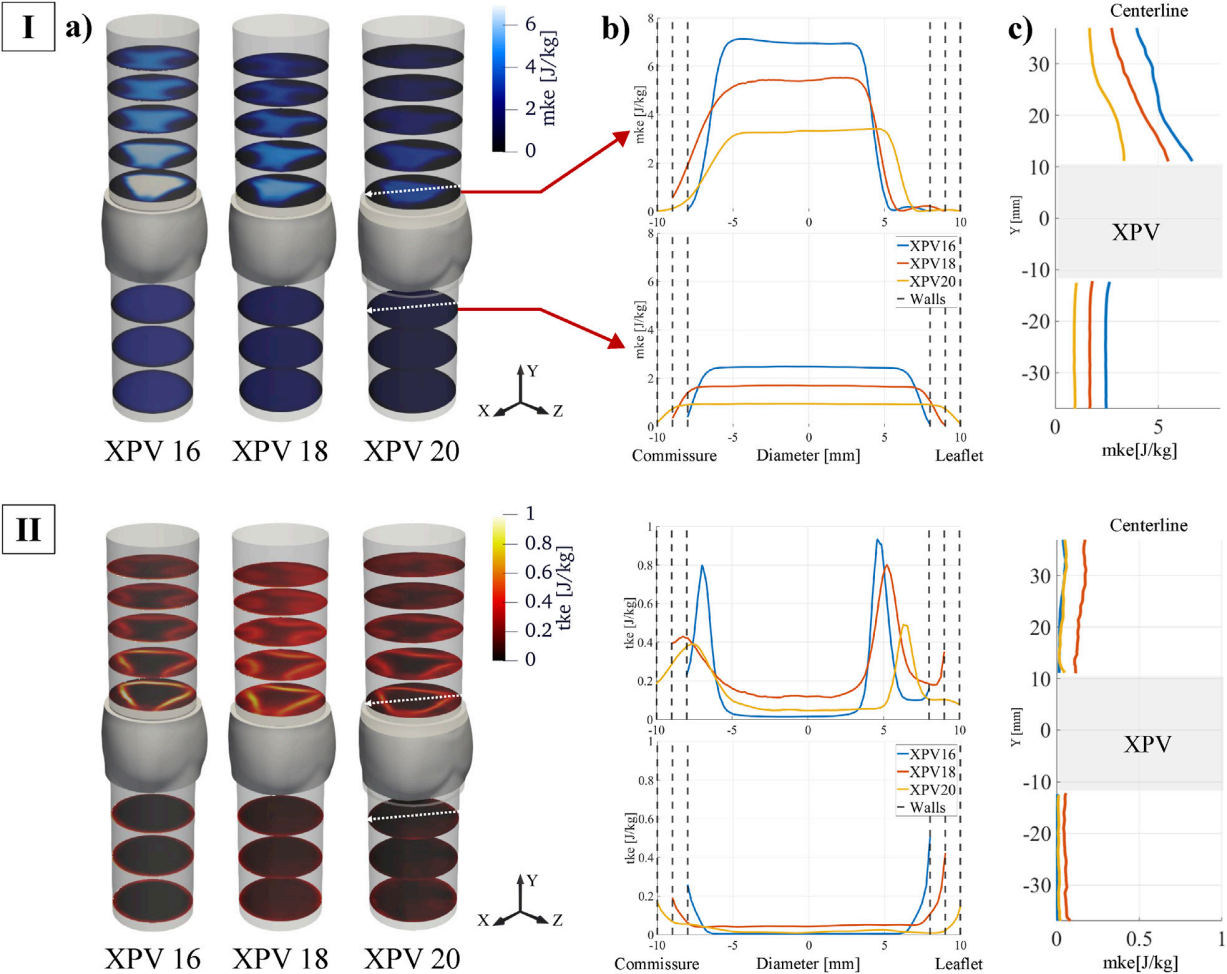
(I) Mean kinetic energy (mke) and (II) turbulent kinetic energy (tke) at peak systole (t_φ_ = 110 ms). **(a)** Quantities displayed at different cross-sectional planes perpendicular to the streamwise direction. **(b)** Quantities along transversal line cutting through one commissure and one leaflet middle axis. The top plot represents the first downstream slice while the bottom plot shows the last upstream slice (indicated by red arrows and by the white arrows on each slice). **(c)** Quantities shown along the centerline.

The spatial distribution of the energy associated with the bulk velocity (*mke*) and its fluctuating component (*tke*) is shown in Figure 5. For all tested XPVs, *mke* exhibited energy levels up to ten times higher than *tke*. The highest *mke* values were observed in the main jet, while regions of elevated *tke* were concentrated in the shear layers. Smaller XPV sizes were associated with higher *mke*: compared to the *mke* levels for XPV20, the 95th percentile of *mke* for XPV16 was 105% higher and 52% higher for XPV18. Across the valve, *mke* increased by 152%, 175%, and 218% for XPV16, XPV18, and XPV20, respectively.

The *tke* levels upstream of the valve were small, except for narrow layers close to the walls, indicating mostly undisturbed inflow. Across the valve, the 95th percentile of *tke* increased by 62% (XPV16), 138% (XPV20), and 161% (XPV20). In the shear layers of the jet *tke* was highest for XPV16 and lowest for XPV20. In the bulk, *tke* was highest for XPV18.

### 3.3 Viscous stresses

**FIGURE 6:**
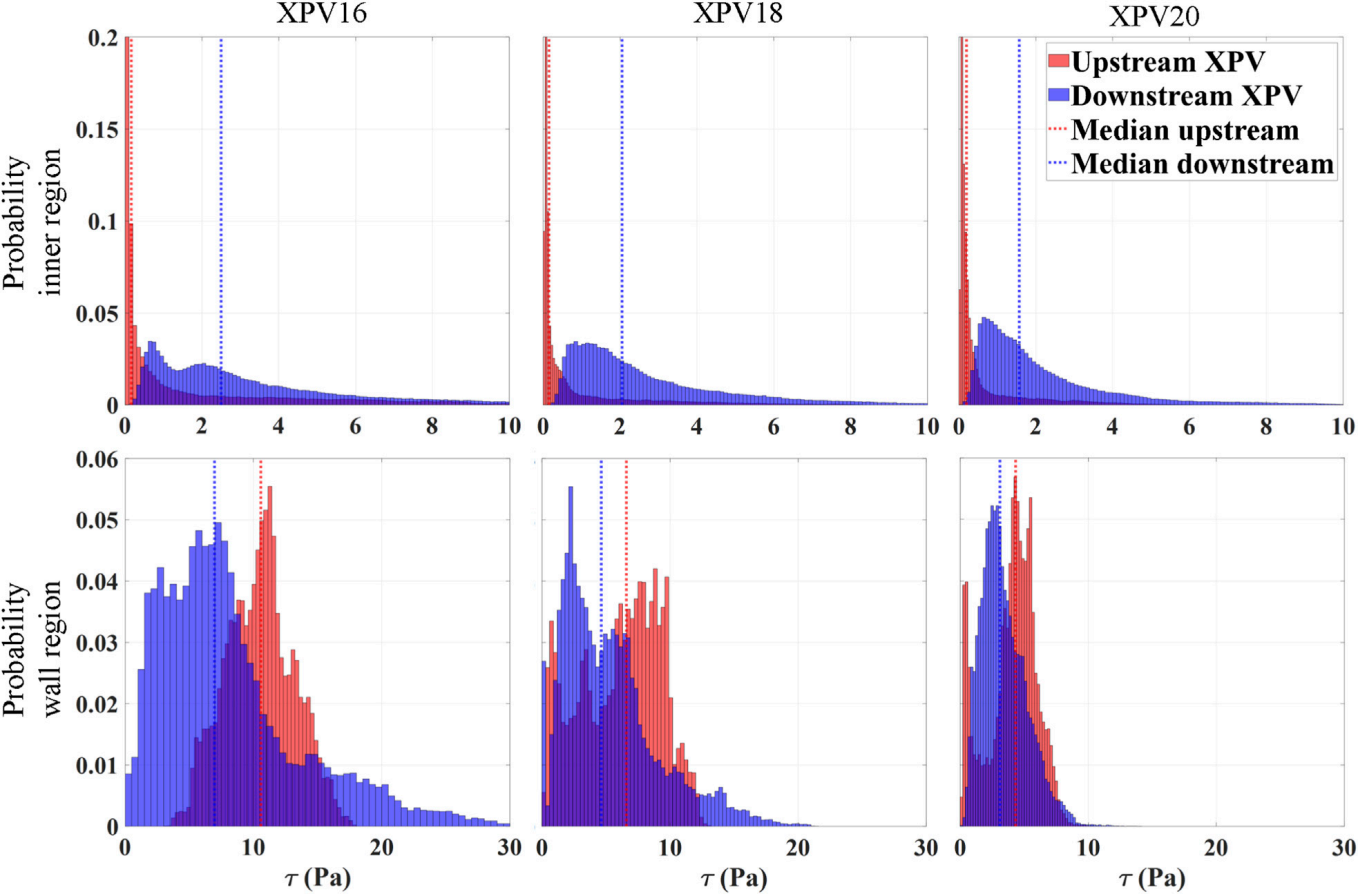
Normalized probability distribution of the shear stresses at peak systole (t_φ_ = 110 ms) divided for the inner region (top) and wall region (bottom). The number of bins is defined according to the Rice rule, with the sum of the heights of all bins equal to 1, representing the total probability across the entire dataset. The median of the distribution is shown as a vertical line. Blue represents values downstream of the valve while red refers to the upstream region.

This section presents viscous shear stresses computed from the reconstructed velocity fields. Although PIV techniques provide reliable velocity measurements across most of the domain, computing shear stresses is difficult due to numerical errors arising in the computation of gradients from velocity fields with limited spatial resolution. Moreover, capturing flow characteristics near the walls remains challenging. The presence of solid boundaries introduces problems such as optical distortions, lack of light, lack of tracers and concerns related to the size of the interrogation window, resulting in reduced accuracy in resolving near-wall velocities (Vamsi Krishna et al., 2020). To account for these limitations, the analysis of shear stresses was divided into two regions. The inner region accounts for most of the fluid domain (bulk flow), excluding a thin layer of 0.5 mm next to the wall. The real numerical values for the viscous stresses are likely somewhat higher than the values reported here. The wall region accounts for the remaining domain (0.5 mm wall layer). The distribution of the maximum coordinate-invariant viscous stress τ is shown as a histogram approximating its probability distribution in Figure 6.

Within the inner region, the stress levels increased significantly as the flow passed through the valve, with the median of the distribution being approximately 16, 13, and 8 times higher for XPV16, XPV18, and XPV20, respectively. Downstream of the valve, the peak stresses were concentrated in the shear layers of the jet, while they were homogeneously distributed over the lumen upstream of the valve. With increasing size, the distribution shifted towards lower stress values with more pronounced peaks and smaller tails toward elevated stress levels. The median stress downstream of the valve was 2.5, 2, and 1.5Pa for XPV16, XPV18, and XPV20, respectively. In the wall region, the stress levels significantly decreased with increasing valve size. The median stress downstream of the valve was 7, 6.6, and 4.3Pa, while 10.6, 4.6, and 3.1Pa were obtained upstream of XPV16, XPV18, and XPV20, respectively.

## 4 Discussion

This study presents the first quantitative evaluation of the velocity fields upstream and downstream of prosthetic valves, which allows for an assessment of the flow downstream of the valve with respect to the inflow boundary conditions. This has not been done in most other experimental studies of valvular flow such that none of these studies were able to determine whether the measured flow field downstream was mainly the result of the specific valve design or whether it was the results of a perturbed inflow.

Furthermore, this study presents a direct comparison of valve grafts of different sizes. Given that the hemodynamic conditions (cardiac output, pressures) are the same for the three valve sizes, we should intuitively expect lower Reynolds numbers, lower velocities, and lower turbulence levels for the larger valve sizes. The presented results confirm these assumptions quantitatively.

In clinical practice, the XPVs are designed as a solution for pediatric patients, with the smaller sizes tailored for younger ages to match the arterial dimension (Akay et al., 2009). The *CO* in this heterogeneous category typically increases with growth (Yoganathan et al., 2013), requiring further investigations on the influence of this parameter on valve performances.

The results of this study demonstrate that the XPV exhibits performance comparable to both established and experimental solutions of equivalent dimensions used for reconstruction of the right ventricular outflow tract (RVOT). The measured pressure gradients were within the same range as clinical data reported for homografts, autologous tissue-valved conduits, and both stentless and stented xenografts. These conduits typically restore a transvalvular pressure gradient (*TVPG*) in the range of 10–25 mmHg, though values can exceed 80 mmHg in cases of implant failure (Yuan et al., 2008). In addition to the *TVPG*, the effective orifice area (*EOA*) of the XPV also aligned with previously reported values from *in vitro* testing of polymeric conduits at a flow rate of 5 L/min. These studies reported TVPGs of 18.8, 19.6, and 26.9 mmHg, with corresponding systolic orifice areas measuring approximately 70% of the conduit diameter (Chang et al., 2020).

### 4.1 Inflow conditions

The flow field upstream of the valves did not show any significant perturbations such that the *tke* levels remained one order of magnitude lower than the *mke* for all valve sizes.

Nevertheless, we found differences in the viscous stresses close to the wall which were highest for XPV16 (median 7.6Pa and 10.6Pa downstream and upstream of the valve, respectively) and lowest for XPV20 (median 4.3Pa and 3.1Pa downstream and upstream of the valve, respectively). This may be attributed to the higher bulk velocities for the smaller valves which led to sharper gradients close to the wall. Despite these differences at the walls, we believe that it is fair to assume that the used experimental setup yielded mostly undisturbed and fully developed inflow boundary conditions for all tested valves. We conclude that the observed differences downstream of the valves were mainly caused by the valves and were not the result of different inflow conditions.

### 4.2 Effect of valve size

While comparable flow conditions were prescribed in all experiments, higher velocities were observed upstream of smaller valves due to the smaller cross-section of the conduit. This difference also led to higher jet velocities in smaller XPV. However, the relative increase in velocity (compared to the inflow velocities) was higher for bigger XPV. This finding suggests that due to the high velocities observed in smaller valves, the leaflets better open towards the sinuses during systole, leading to differences in leaflet kinematics and structural stresses distribution. Further analysis performed with high-speed cameras is required to better understand the potential implications on leaflet motion and long-term valve performance.

The relative increment of *mke* was higher in the smaller XPV, while the less constrained jet observed in bigger size resulted in a broader but lower peak values of *tke.*

The analysis of viscous shear stresses was divided into wall and inner regions, with the latter being representative of the stresses induced by the main jet issued from the valve orifice. In this region, the levels of *τ* at peak systole for all XPVs were one order of magnitude below the critical hemolytic threshold of 150–250Pa (Brown et al., 1975; Sutera and Mehrjardi, 1975; Colantuoni et al., 1977), while fewer than 5% of the shear stresses reached levels associated with potential platelet activation of 10–30Pa (Hung et al., 1976; Ramstack et al., 1979). However, these critical stress levels refer to an exposure time of more than 100 s which is several orders of magnitude longer than the whole cardiac cycle. Therefore, these values cannot be used for a quantitative assessment of the blood damage potential.

The interpretation of the results in the wall region remains more challenging given the limitation of tomo-PIV at the walls. Nevertheless, we found that downstream of the valve, the stress distribution becomes broader and reaches higher stress values, which reflects the influence of the jet profile. On the one hand, the regions associated with the corners of the triangular-shaped jet were characterized by a sharp deceleration to meet the no-slip conditions at the wall and were associated with increased shear stresses. On the other hand, the regions of retrograde flow (between the corners) were associated to smoother velocity gradients than in the corner regions, accounting for lower stresses. In conclusion, shear stress levels at peak systole increased as the XPV size decreased, with an overall probability of 6% (XPV16), 2% (XPV18), and less than 1% (XPV20) of encountering elevated viscous stresses potentially relevant for platelet activation.

These results indicate that the choice of a larger size is preferable when there are no physiological restrictions. Implanting an undersized valve, relative to the native physiological dimensions, can lead to abnormal flow patterns and elevated level of blood stresses, both of which contribute to impaired hemodynamics and a heightened risk of thrombogenic complications.

### 4.3 Limitations

The silicone phantoms were designed to replicate the original geometry of the XPV conduits while providing the optical access necessary for particle tracking and velocity reconstruction. Although the phantoms matched the optical properties of the testing fluid, they did not accurately replicate the porosity or mechanical properties of the actual conduits. Furthermore, a single specimen was investigated per size, such that manufacturing variability was not addressed in this study. While this study presents a baseline of valve performance for different dimensions, inferential statistical analyses such as ANOVA or t-tests were not feasible, as they require multiple independent samples per group.

The use of a single specimen in studies of valve hemodynamics is a common practice when testing mechanical or biological valves (Pietrasanta et al., 2022; Ferrari and Obrist, 2024; Chen and Dasi, 2023; Sturla et al., 2020), due to their highly standardized and reproducible manufacturing processes. While the Xeltis tissue-engineered valves used in this study are also produced under controlled and repeatable conditions, tissue-engineered scaffolds in general may be subject to greater variability in structure and material properties across samples. Therefore, although our results are representative of the specific specimen tested, they may not fully capture potential inter-sample variability that could exist in other contexts or production batches.

In clinical practice, tissue-engineered (TE) valved conduits are typically primed with blood prior to implantation to enhance leaflet permeability. While the tested XPVs were not subject to any pre-treatment and only consisted of the decellularized scaffold, data collected during diastole showed that all valves were sealed as no backflow was observed. It is important to note that TE valves experience structural changes over time due to cellular proliferation and scaffold degradation. Therefore, the results of this study should be interpreted as representative of the post-implantation scenario. However, the goal of this study was not to assess the clinical outcomes of different XPV sizes but rather to provide a systematic comparison of the hemodynamic characteristics across different valve sizes.

Previous studies on biological valves tested under similar conditions reported Kolmogorov length scales on the order of 100 *µ*m or less. In the present study, limitations in spatial resolution and the usage of a phase-locked approach could potentially lead to an underestimation of the measured viscous shear stresses.

## 5 Conclusion

The present experimental study evaluated the influence of valved conduit sizes on the flow characteristics upstream and downstream of the valve samples by applying the same test conditions to three dimensions of XPVs. Higher flow velocity and transvalvular pressure gradients were associated with smaller valve sizes. Smaller valve sizes were linked to a higher probability of encountering elevated shear stresses downstream of the valve, which could have implications for platelet activation and long-term valve performance.

We provide a systematic comparison of valve sizes, advancing our understanding of flow dynamics in tissue-engineered valves and guiding future *in-vitro* investigations. Additionally, we present the first *in-vitro* experimental evaluation of the XPV valve alongside a detailed investigation of valve size effects, ensuring that these findings can be translated across various biological and polymeric valve models. This study does not address potential inter-sample variabilities, which may be more relevant for novel approaches such as tissue-engineered valves. Therefore, future work should extend the analysis to more samples to provide inferential statistical analysis and strengthen our conclusions.

## Data Availability

The raw data supporting the conclusions of this article will be made available by the authors, without undue reservation.

## Abbreviations

CO, Cardiac output; CC, Compliance chamber; CHDs, Congenital heart diseases; EOA, Effective orifice area; ETR, Endogenous tissue restoration; mke, Mean kinetic energy; PP, Piston pump; PA, Pulmonary artery; ROI, Region of interest; RIM, Refractive index matched; Re, Reynolds; RA, Right atrium; RVOT, Right ventricular outflow tract; STB, Shake-The-Box; STJ, Sinotubular junction; Tomo-PIV, Tomographic particle imaging velocimetry; TVPG, Transvalvular pressure gradient; TV, Tricuspid valve; TR, Tunable resistance; tke, Turbulent kinetic energy.
